# Massive Haematochezia due to Splenic Artery Bleeding into the Colon: Unusual Manifestation of Advanced Pancreatic Cancer

**DOI:** 10.1155/2023/7443508

**Published:** 2023-01-12

**Authors:** R. Sguinzi, F. Pugin, C. Bader, A. Meyer, L. Buhler, L. Widmer, D. Staudenmann, B. Egger

**Affiliations:** ^1^Department of General Surgery, Fribourg Cantonal Hospital, Fribourg 1700, Switzerland; ^2^Faculty of Science and Medicine, Section of Medicine, University of Fribourg, Fribourg 1700, Switzerland; ^3^Department of Diagnostic and Interventional Radiology, Fribourg Cantonal Hospital, Fribourg 1700, Switzerland; ^4^Praxis Intesto, Department of Gastroenterology, Fribourg Cantonal Hospital, Fribourg 1700, Switzerland

## Abstract

We describe a case of an uncommon early pancreatic cancer presentation in a patient in his 60s who had haemorrhagic shock from extensive haematochezia and required blood transfusions as well as surveillance in an intensive care unit. A splenic artery pseudoaneurysm that had been effectively embolized by angiography was seen to be actively bleeding into the colon lumen on a computerized tomography (CT) scan along with a necrotic mass of the pancreatic tail. A pancreatic mucinous adenocarcinoma was diagnosed by a transgastric biopsy. A pancreatico-colic fistula was discovered by CT scan after a colic contrast enema. A transabdominal drainage of the necrotic collection and targeted antibiotic treatment had been performed with a satisfying patient outcome. In order to assess a potential secondary surgical resection, systemic chemotherapy was planned. In conclusion, haematochezia with hemodynamic instability originated from a splenic artery pseudoaneurysm fistulising into the colon (arterio-colic fistula) and sepsis originating from a tumoral pancreatic abscess fistulising into the colon (tumoral pancreatico-colic fistula).

## 1. Introduction

Pancreatic ductal adenocarcinoma (PDAC) is the seventh leading cause of cancer-related mortality [1] and is associated with a five-year survival rate around 10% [2]. PDAC is a relatively uncommon cancer, with approximately 60,430 new diagnoses expected in 2021 in the United States. By 2030, PDAC, which now ranks as the second biggest cause of cancer-related mortality, is expected to have an annual incidence rate of 0.5–1.0%. Compared to 5.26% in 2000, the 5-year survival rate reached 10% for the first time in 2020 [3].

The majority of tumors (about 70%) develop in the head of the pancreas and frequently present with biliary obstruction, which results in dark urine (49%), jaundice (49%), appetite loss (48%), fatigue (51%), weight loss (nearly 55%), and exocrine pancreatic insufficiency (25%) [4].

The majority of pancreatic malignancies (90%) are PDACs and their variations, with 60–70% of cases occurring in the head, 15% in the body, and 15% in the tail of the pancreas [4, 5]. When situated in the tail, they tend to invade fewer structures due to their anatomical localization and, therefore, display less symptoms leading to an advanced stage at diagnosis.

Tumor local invasion is based on its resectability and falls into three categories: resectable, borderline resectable, and unresectable. The latter are divided into two subcategories: locally advanced (LA) and metastatic.

The tumor is considered LA if: the degree of contact or invasion of the superior mesenteric vein, portal vein, superior mesenteric artery, or celiac artery (CA) is equal to or exceeds 180°; the contact or invasion of the common hepatic artery shows contact or invasion of the proper hepatic artery and/or CA; and the aorta is in contact or invaded by the tumor [6].

Therefore, given the close anatomical relationship between the tail of the pancreas and the blood vessels of the spleen, the latter is likely to be involved during the progression of pancreatic tumors of the body or tail of the pancreas [7] leading in a development of a splenic artery (SA) pseudoaneurysm due to digestion of the external wall by pancreatic enzymes [8]. By the same adjacency principle, a pancreatico-colic fistula may arise from the transverse colon or colonic left flexure through the transverse mesocolic ligament with an incidence of 1.4–4% [9, 10]. Two cases of pancreatico-colic fistula formation secondary to pancreatic masses, adenocarcinoma, and intraductal papillary mucinous tumors have also been reported in the literature [11, 12].

We report a case of pancreatic cancer presenting with haematochezia originating from a splenic artery pseudoaneurysm rupture into the colon lumen.

## 2. Case Presentation

**Figure 1:**
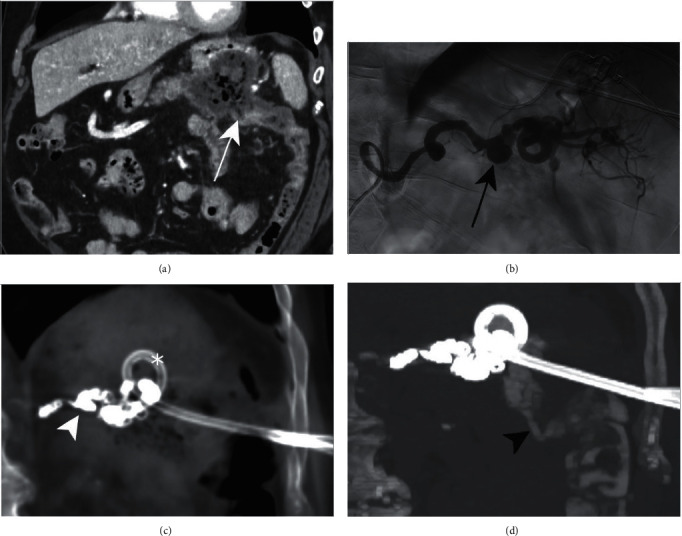
(a) Coronal reformatted contrast-enhanced CT demonstrates a large necrotic tumor in the pancreatic tail with suspicion of a fistula extending to the left colic angle (white arrow). (b) Digital subtraction angiography of the splenic artery shows caliber irregularities of the proximal and middle thirds of the vessel and a 15 mm diameter pseudoaneurysm (black arrow) of the middle third of the artery, without contrast extravasation; in the normal segments, the splenic artery measures 4–5 mm of diameter. Coronal reformatted CT with maximal intensity projection before (c) and after (d) positive contrast injection through the drainage catheter (asterisk) placed in the necrotic pancreatic mass reveals a pancreatico-colic fistula (black arrowhead) responsible for a concomitant opacification of the left colon. Images were acquired after endovascular embolization with coils (white arrowhead).

A 64-year-old male patient presented to our emergency department after five episodes of haematochezia associated with episodes of vomiting without other symptoms in the past 9 hours. At admission, Glasgow Coma Scale (GCS) was 15/15, heart rate was 105 beats/minute, blood pressure was 100/71 mmHg, respiratory rate 28 breaths/minute, SO_2_ 93%, temperature 36.7°C, pain evaluation 0/10, and Body mass index (BMI) 38 kg/m^2^. Abdominal examination showed diffuse tenderness with no palpable mass or peritonitis. Patient has a personal history of coronary artery disease with a diagnosis of Non-ST-elevation myocardial infarction (NSTEMI) treated with coronary stents and aspirin, recurrent superficial venous thrombosis, diagnosed 1 year ago, treated with rivaroxaban for 3 months, metabolic syndrome with dyslipidemia, hypertension, type 2 insulin-dependent diabetes mellitus, obesity, and active smoking at 40 pack year. No family history was discovered. Blood tests showed a haemoglobin value of 121 g/L at first check and 78 g/L 12 hours later, justifying the transfusion of 4 units of blood and 1 plasma concentrate. Three phase computerized tomography (CT) scan showed a hypodense air-containing mass measuring 8 cm × 8 cm × 7 cm in the tail of the pancreas suggestive of a necrotic tumor lesion, with infiltration of the colon splenic flexure and fistula (Figure 1(a)). An irregular caliber of the SA in contact with the posterior part of the mass and infiltration of the gastric wall was reported; multiple enlarged retroperitoneal lymph nodes were also present. Immediate esophagogastroduodenoscopy and colonoscopy did not find active bleeding, but showed a small amount of blood in the bulb of the duodenum and a small amount of melena throughout the colon. The patient was monitored in the intensive care unit, and transfused 2 more units of blood and 1 unit of fresh frozen plasma. Hypotension after a new episode of haematochezia motivated a new injected CT scan that showed a 15 mm pseudoaneurysm of the splenic artery, with a normal caliber of the splenic artery of 4–5 mm, in contact with a pancreatic mass with an arterio-colic fistula. Radiologic and clinical Tumors-Nodes-Metastases staging of the tumor was cT3 cN1 cM0, stage IIB according to Modified 8th American Joint Committee on Cancer (AJCC). Echoendoscopy showed a 45 mm solid hypoechoic mass invading the SA with necrotic and cystic areas in the tail of the pancreas. A diagnosis of pancreatic mucinous adenocarcinoma was made by fine-needle aspiration, and immunohistochemical staining was positive for CK7/116, CK20, and CDX2. A polypoid lesion of the greater curvature of the stomach was also biopsied, showing pancreatic tumor infiltration. Carcinoembryonic antigen (CEA) is 43.9 ng/ml (normal: <3.0 ng/ml), and CA 19-9 is 1,070 U/ml (normal: <35 U/ml). Microbiological analysis showed the presence of *Enterococcus faecalis, Enterococcus avium*, and *Candida albicans.*

Therapeutic angiography was performed, confirming the formation of a middle third splenic artery pseudoaneurysm without active bleeding on angiography (Figure 1(b)). Embolization of the pseudoaneurysm was accomplished by endovascular coiling, allowing the patient to be observed in the surgical department. Targeted antibiotic therapy with piperacillin/tazobactam and fluconazole was administered for 1 month. The embolization procedure was complicated by splenic necrosis and sepsis due to secondary infection with multidrug-resistant *Staphylococcus epidermidis*. After an unsuccessful endoscopic attempt to drain gastric contents due to thickened necrosis, CT-guided percutaneous drainage was performed with the insertion of a 10 F drainage catheter, upsized with a 20 F catheter. Antibiotic therapy was discontinued after 1 month as the collection was completely emptied and the patient's condition stabilized. A neoplastic pancreatic abscess fistulising into the colon (neoplastic pancreatico-colic fistula) was confirmed by CT imaging after positive contrast injection into the catheter (Figures 1(c) and 1(d)). On hospital day 11, the patient developed acute dyspnoea with evidence of hemodynamic shock and was diagnosed with bilateral central pulmonary embolism by contrast-enhanced thoraco-abdominal CT scan. The patient was transferred again to the intensive care unit for hemodynamic stabilization and management. Bedside transthoracic echocardiography revealed a severe right ventricular dysfunction motivating local thrombolysis and the introduction of therapeutic doses of intravenous heparin. Angiological examination revealed deep vein thrombosis in the right leg. Controls for subsequent abdominal images were unchanged.

Patient outcome was subsequently favourable. An interdisciplinary consensus for evaluating future surgical and oncological plans advocates systemic neoadjuvant chemotherapy with nab-paclitaxel and gemcitabine as first-line treatment 3 months after initial hospitalization. Furthermore, evaluation will be done after 3 months to plan for the possibility of multi-organ resection, but hemodynamic instability due to bleeding, accumulation of infection, and LA neoplasms make it a non-first-line treatment. The patient was discharged in stable condition with drainage in place. At the start of chemotherapy treatment, as the disease progressed, the patient developed colic obstruction at the level of the tumor mass, which caused a compressive effect. A colostomy was performed because the colic dilation was 56 mm in the transverse and 95 mm in the cecum. The patient was newly admitted to the emergency department in the context of haemorrhagic shock, after two episodes of hematemesis. The CT scan showed the development of a fistula between the tumor and the stomach, thus presenting a gastro-pancreatic fistula with intraluminal arterial bleeding from the distal third of the transverse colon and from the left colic angle, with a possible source of bleeding from a branch of the left colic artery. Gastric fistula explains hematemesis. After discussion with the family and the patient, the decision to discontinue treatment and care for comfort was made. The patient died 6 months after the initial diagnosis.

## 3. Discussion

Massive haematochezia due to splenic artery pseudoaneurysm rupture, fistulised into the colon, is a potentially lethal condition, which requires prompt diagnosis and treatment and a potentially lethal and challenging condition [13]. A massive gastrointestinal tract bleeding of unknown etiology should raise diagnostic suspicion of pancreatic disease, especially in patients with a history of acute or chronic pancreatitis or pseudocyst [14, 15].

The most common evolution of pancreatic necrosis is abscess formation [16] generating internal fistulas extending to the pancreas and potentially to the colon, stomach, duodenum, jejunum, ileum, pleura, or bronchial system. The development of pseudoaneurysm and bleeding secondary to rupture are the result of vascular erosion. The most common site is the splenic artery, but any peripancreatic vessel may be involved. The incidence of splenic artery pseudoaneurysm is low, but should be suspected in patients with persistent pancreatitis in whom abdominal mass suddenly disappears or gastrointestinal bleeding develops due to fistula at the splenic angle of the colon [17]. A pancreatic tumor that behaves like necrotizing pancreatitis should also be considered in the differential diagnosis.

The initial approach often requires multimodality imaging to properly depict the extent of the disease. Contrast multiphase abdominal CT can identify both active bleeding due to ruptured pseudoaneurysm and signs of a malignant fistula extending into the colon. It should be noted that positive contrast media should not be used as it will not reveal the passage of vascular contrast medium into the colon through the fistula. The precise localization of the source of bleeding can be achieved by selective angiography of the visceral arteries. Arteriography with selective embolization of the bleeding artery is the best treatment in emergency phase [15].

In case of refractory sepsis, surgical colon resection or derivation may be mandatory.

Endoscopic procedures may also take place in the treatment, even if with some limits. In case of a pancreatic collection with complete necrosis of the neck or body of the pancreas, endoscopic retrograde cholangiopancreatography could be difficult as the pancreatic gland integrity is rarely preserved and may lead to superinfection. Endoscopic sphincterotomy and placement of a short transpapillary stent may reduce intraluminal pressure and associated inflammation, promote healing, and repair ductal discontinuities [18]. When this type of drainage is inadequate, Endoscopic-Ultra-Sound-guided transabdominal drainage using a double-pigtail stent is the gold standard in patients with disconnected pancreatic duct syndrome [19, 20].

Other approaches may be considered in certain circumstances. If immediate surgical indication is not possible, as in the case shown here, the colic fistula can be closed with endoscopic clips [21, 22]. Large, rapidly enlarging, obstructive, or infected collections require image-guided percutaneous drainage of peripancreatic collections [23].

The timing of surgery is critical to achieve oncological R0 resection. Hemodynamic stability should be restored, and the septic condition should be treated first. Oncologic staging should evaluate the resectability, and treatment options should be discussed in a multidisciplinary team meetings. Neo-adjuvant chemotherapy may increase the rate of R0 resection in cases of borderline or LA PDAC [24–26].

In conclusion, advanced pancreatic cancer with massive haematochezia can occur secondary to neoplastic pancreatico-colic fistula and ruptured splenic artery pseudoaneurysm, and requires prompt diagnosis and multimodal therapy. Treatment options must be weighed according to several factors, and the risk of morbidity and mortality remains high.

## Data Availability

Data supporting this research article are available from the corresponding author or first author on reasonable request.
